# The Associations among Destructive Leadership, Job Demands and Resources, and Burnout among Nurses: A Cross-Sectional Survey Study

**DOI:** 10.1155/2023/4289450

**Published:** 2023-12-28

**Authors:** Tuuli Palvimo, Anneli Vauhkonen, Marja Hult

**Affiliations:** ^1^Department of Nursing Science, University of Eastern Finland, Kuopio, Finland; ^2^Orton Orthopaedic Hospital, Tenholantie, Helsinki, Finland

## Abstract

**Background:**

Nurses demonstrate high burnout prevalence. Moreover, destructive leadership, as well as job demands and resources, are associated with burnout. However, these associations, particularly in the context of nursing, warrant further investigation.

**Objective:**

To explore the associations of destructive leadership, as well as job demands and resources, with burnout in registered nurses.

**Design:**

A cross-sectional survey. *Participants*. 2115 registered nurses in Finland.

**Methods:**

The self-administered questionnaire survey was distributed nationwide to 106,000 members of the Finnish trade union for health and social care professionals via an online newsletter in February 2023. Nurses' burnout was measured with the Burnout Assessment Tool (BAT). The data were analysed through descriptive statistics and linear regression analysis.

**Results:**

Destructive leadership and job demands were positively associated with burnout (*β* = 0.39 and 0.32, respectively; both *p* < 0.001), whereas job resources and burnout were negatively associated (*β* = −0.41, *p* < 0.001). The associations of destructive leadership and job demands with burnout became less positive when job resources were added to the regression model (*β* = 0.21 and 0.14, respectively; both *p* < 0.001).

**Conclusions:**

Job resources led to the greatest reduction in burnout among registered nurses. Moreover, job resources reduced burnout by diminishing the negative effects of destructive leadership and job demands. Providing a sufficient amount of job resources might reduce burnout and diminish the negative effects of destructive leadership and job demands among nurses. These relationships warrant examination in other cultural settings.

## 1. Introduction

The global shortage of the healthcare workforce, particularly nurses, is a major issue; the World Health Organization (WHO) projects that this shortage will reach 5.7 million by 2030. Population ageing has led to an increase in the demand for health services. However, the nursing workforce is ageing as well; approximately, one of six nurses is expected to retire by 2030 [1]. Other challenges, including the COVID-19 pandemic, have exacerbated these issues. Working in healthcare is stressful; furthermore, the nursing staff work under extreme pressure, which can lead to health problems, such as burnout, and to insufficient personnel retention and recruitment [2, 3]. Therefore, facilitating mental health improvement among nurses through the improvement of their demanding working conditions is crucial. To support European countries with severely burdened healthcare systems, the WHO proposed 10 effective policy and planning responses; one of them is “protect the health and mental well-being of the workforce” [2].

Complex burnout syndrome, which most commonly results from prolonged exposure to work-related stress, is characterised by the dimensions of exhaustion, cynicism, and inefficacy [4]. In particular, overwhelming exhaustion is often the first sign of work-related health issues. Over time, it leads to detachment and withdrawal from work, as well as cynicism, followed by negative self-evaluation and the feeling of incompetence. The role of a few of these dimensions, particularly inefficacy, in burnout development has been re-examined; as such, the definition of burnout has been revised [5, 6]. Schaufeli et al. [6] indicated four core elements of burnout, namely, exhaustion, mental distance, emotional impairment, and cognitive impairment. Exhaustion refers to an extreme lack of physical and mental energy, which reduces an individual's capacity to regulate emotions (e.g., anger and sadness) and cognitive processes (e.g., memory and attention). This results in the development of a coping strategy involving mental withdrawal and detachment from the job. In a Delphi study, an expert panel unanimously defined burnout as prolonged work-related exhaustion [7]. Exhaustion, generally considered the most essential dimension of burnout, is included in most burnout questionnaires [6].

The average burnout prevalence among nurses varies in different studies, ranging from 11% [8] to 52% [9]. The studies show differences between geographical regions and specialties [8] and educational levels [9]. The review by Ge et al. found a prevalence of 30% and indicated an increased trend over time [10]. However, the accurate estimation of this prevalence can be challenging because appropriate diagnostic criteria for burnout are unavailable [11] and burnout is not considered a medical condition in the International Classification of Diseases 11th Revision [12].

Burnout has several physical, psychological, and occupational consequences on workers' health and well-being; these consequences include type 2 diabetes, coronary heart disease, various types of pain and injuries, insomnia, job dissatisfaction, absenteeism, and the need for disability pension [13]. In addition, a positive association between burnout and depression among nurses has been found [14]. Furthermore, higher burnout levels appear to lead to a stronger occupational turnover intention [15–17]. Burnout-attributed turnover and burnout result in a major financial burden on organisations and societies [18–20]. Moreover, nurse burnout affects perceived patient safety, satisfaction, and quality of care [21]. Therefore, burnout is a severe health issue with individual, organisational, and societal consequences [22].

The job demands-resources (JD-Rs) model was originally tested in a study on German nurses and subsequently developed into a theory [23–25]. According to this model, different physical, psychological, social, or organisational aspects of a job, particularly if they are imbalanced, can affect employee health outcomes. Job demands, such as a high workload, require sustained effort and are associated with exhaustion. Job resources are job aspects that support the achievement of work goals and personal growth and development, reduce job demands, and protect from burnout; examples of these resources are autonomy and job security. In the JD-R model, resource insufficiency is associated with disengagement [24]. In the current study, we used the JD-R model as the theoretical framework.

The effect of job demands and resources among nurses have been studied comprehensively, and the association of job demands with burnout has been established [22, 26–28]. Among nurses, a high workload is also correlated with decreased patient safety and quality of care [26]. In contrast, job resources are negatively associated with burnout [29, 30]; they may buffer the effects of job demands on strain [31, 32] and burnout [33].

A major determinant in work-related health outcomes in nurses is leadership, and the favourable effects of positive leadership styles are well known [34–36]. Recent management research has focused on adverse leadership styles and their effects on followers and organizations. One such adverse leadership style is the so-called destructive leadership style [37, 38].

Destructive leadership is a process involving a supervisor's systematic and repeated hostile behaviour or incompetence as perceived by their followers or subordinates. Destructive leadership can be intentional or unintentional and physical or verbal and has harmful consequences on the well-being, job satisfaction, and performance of the workers, and the goals of the organisation, or both [37, 39, 40]. Most empirical studies on destructive leadership have examined abusive supervision using Tepper's [41] abusive supervision scale [38]. In contrast to destructive leadership, abusive supervision excludes physical contact [41] and aims to control workers by creating fear and intimidation [39]. Furthermore, under toxic leadership, a leader's destructive behaviour or dysfunctional personal qualities have a debilitating effect on their followers [42]. In the toxic triangle model, toxic leadership is derived from the interaction between the leader, their followers, and the conducive environment [43]. Petty tyranny is described as authoritarian behaviour where a leader uses power oppressively, capriciously, and vindictively over their subordinates [44]. Einarsen et al. [39] categorised the leadership styles as tyrannical (including humiliation, belittling, and aggression), derailed (including bullying, manipulation, harassment, absenteeism, and shirking), supportive -disloyal, and constructive; these behaviours can have destructive and constructive effects on different dimensions simultaneously.

Destructive leadership is positively related to nurses' psychological strain, burnout, and intention to quit the job or leave the profession [31, 35, 45]. It is also associated with decreased work effectiveness and performance [46] and nurse-reported adverse events and quality of care [31, 47]. Strengthening nursing and midwifery leadership is one of the policy priorities of the WHO Global Strategic Directions for Nursing and Midwifery 2021–2025 [48].

Despite increasing scientific interest in destructive leadership, knowledge regarding this detrimental phenomenon, particularly in nursing, remains limited [36]. Thus far, the associations of destructive leadership, as well as job demands and resources, with burnout among nurses have not been explored. By gaining further insight into the roles of leadership and working conditions in burnout development among nurses, healthcare organisations may be able to develop and implement burnout prevention strategies. In the current study, we explored the associations of destructive leadership and job demands and resources with burnout among registered nurses in Finland. We also analysed burnout by different background characteristics. Our results may clarify the need for leadership development and education for maximum staff retention.

## 2. Methods

### 2.1. Study Design

This was a cross-sectional study conducted in accordance with the STROBE guidelines for cross-sectional studies [49].

### 2.2. Data Collection and Participants

The data were collected over a 3-week period in February 2023 by using an online self-administered questionnaire. Participants were recruited via an online newsletter of a Finnish trade union for health and social care professionals, distributed to 106,000 of its members. The sampling method can be, therefore, called purposive sampling. We also sent a weekly reminder to every member, requesting them to respond to the questionnaire. In total, 4575 responses were received; of them, 2370 were from registered nurses. After respondents who performed supervisor tasks (*n* = 247) were excluded, the final sample size was 2115. Considering the survey invitation and distribution methodology, the accurate survey response rate could not be calculated.

### 2.3. Measurement Tools

The survey collected the following background characteristics: age, gender, education level, work experience (years), and workplace. Moreover, three scales were used to measure burnout, destructive leadership, and job demands and resources.

#### 2.3.1. Burnout

Burnout was measured using the 4-item Burnout Assessment Tool (BAT-4) [50]—a shortened version of the original 23-item BAT (BAT-23) [6]. BAT-23 was developed to assess the four core dimensions of burnout, namely, exhaustion, mental distance from work, cognitive impairment, and emotional impairment. The BAT-23 can be applied based on group or individual assessments [6]; its applicability for the cross-country comparison of burnout has also been reported [51]. The BAT-23 has been validated and noted to have good psychometric properties; moreover, both the 12-item BAT (BAT-12) and the BAT-4 have shown to reliably explore the dimensions of burnout. The BAT-4 is strongly correlated with the BAT-12 (*r* = 0.94) and BAT-23 (*r* = 0.92), and its Cronbach's *α* is 0.73 [50, 52]. In the BAT-4, each item assesses one core dimension as follows: exhaustion (“at work, I feel mentally exhausted”), mental distance from work (“I struggle to find any enthusiasm for my work”), cognitive impairment (“at work, I have trouble staying focused”), and emotional impairment (“at work, I am unable to control my emotions”). Here, the frequency-based responses are scored on a 5-point Likert scale—ranging from 1 (never) to 5 (always); a higher score is considered to indicate more burnout [50].

#### 2.3.2. Destructive Leadership Scale

Destructive leadership was measured based on nine statements related to the employee's relationship with the immediate supervisor as follows: authoritarian leadership (e.g., “I am treated in an authoritarian (i.e., bossy or commanding) way”), abusive leadership (e.g., “I am treated in an unfair or discriminatory way”), and aggressive leadership (e.g., “I am treated in an aggressive manner”). Similar questions have been used to measure the vulnerability caused by management as a dimension of precarious employment (e.g., [53]). The responses were scored on a 5-point Likert scale—ranging from 1 (completely disagree) to 5 (completely agree); a higher score was considered to indicate a higher prevalence of destructive leadership.

#### 2.3.3. Job Demands and Resources Scale

Job demands and resources were measured using items that assess working conditions; these items have been widely used in surveys such as the European working conditions surveys [3]. The scale included the following 18 statements: 8 describing job demands and 10 describing job resources. Statements on job demands included those related to time pressure (“I have to work really fast” and “I have to work hard”), physical workload (“my job requires a lot of physical effort,” “I often have to lift or move heavy loads,” and “I have to work for long periods in uncomfortable positions”), decision-making opportunities (“I have few opportunities to decide how I do my work”), and insecurity (“I have no certainty about the future of my job” and “there is a danger that I will soon lose my job”), whereas statements on job resources include those related to participation in decision-making (“I have the opportunity to make a lot of decisions about my work” and “I have a say in many at my job”), task variety (“I am given a lot of different tasks” and “I have the opportunity to learn new things at my job”), professionalism (“my job requires a high level of professionalism”), the amount of time (“I have enough time to do my work”), work (“I do not have an unreasonable amount of work”), and security (“I could quickly get a new job if I wanted to”). The responses were scored on a 4-point Likert-type scale—ranging from 1 (completely disagree) to 4 (completely agree); a higher score was considered to indicate more job demands or resources.

### 2.4. Data Analysis

Descriptive statistics of all variables were calculated as frequencies and percentages, as well as means and standard deviations (SDs). Missing value analysis demonstrated low rates between 0.01% and 0.4% per scale; it was the highest at 2.0% for the variable of age. For each scale, the missing values were replaced with their respective mean values. The mean values for the study variables were calculated, and ANOVA was used to compare the average levels of burnout in terms of age, education level, and work experience. Independent sample *t* tests were used with dichotomous gender variables. A *p* value of <0.05 was considered to indicate statistical significance.

Scale reliability was analysed by calculating Cronbach's *α*, and the relationships between the variables were calculated as Pearson's correlation coefficients (Table 1). Multicollinearity of the data was also assessed. Furthermore, linear regression analysis was used to explore the associations of burnout with destructive leadership, as well as job demands and resources. All models were adjusted for age, gender, and work experience. The assumptions of homoscedasticity and linearity of the models were tested and confirmed to not have been violated [54]. All statistical analyses were conducted using SPSS (version 27).

### 2.5. Ethical Considerations

There was no need for ethical approval; however, the Institutional Review Board of the participating trade union approved the study [55]. Our questionnaire cover letter included information regarding the study, and it indicated that by answering the questionnaire, the participants provided their informed consent. The questionnaire also included a privacy statement. No personal identification data were collected, and the data were stored on the university's secure server, protected by username and password. The data will be deleted three years after the end of the study.

## 3. Results

As listed in Table 1, the mean participant age was 46 years (range: 22–67 years). A majority of the participants were women (93%), had a bachelor's degree (64%), had >10 years of work experience (66%), and worked in the public sector (86%); of all the participants, 57% worked in hospitals, 21% worked in health and welfare centres and clinics, 13% were social service workers, 3% provided at-home services, 2% worked in private companies or were self-employed, and 4% provided other services such as state- or organization-level, or student, health services.

Table 2 presents the overall BAT scores as well as their distribution among different age, gender, education level, and work experience groups. The mean (SD) total BAT-4 score was 2.62 (0.64), with the score in the dimension of exhaustion being the highest (mean = 3.21 and SD = 0.86). Nurses aged <35 years demonstrated the highest BAT-4 scores, whereas those aged >50 years showed the lowest (*p*=0.003). Women reported significantly higher scores than men (*p*=0.038). The mean scores among nurses with work experience of <5 and >10 years were similar; however, after post hoc correction, nurses with work experience of 5–10 years demonstrated the highest scores (*p*=0.014). No significant differences were noted between the different education level groups.

The results of the destructive leadership scale are presented in Table 3. The statement “my bosses make me feel like I am easily replaceable” demonstrated the highest score (mean = 2.76 and SD = 1.35), followed by “I am treated in an authoritarian (i.e., bossy or commanding) way” (mean = 2.57 and SD = 1.23). However, “I am treated in an aggressive manner” demonstrated the lowest score (mean = 1.57 and SD = 0.94).

On the job demands scale, the highest scores were demonstrated by the statements related to having to work hard (mean = 3.23 and SD = 0.72) and quickly (mean = 3.01 and SD = 0.78). The respondents also reported low job autonomy (i.e., having only few opportunities to decide how they perform their work; mean = 2.57 and SD = 0.78). In contrast, the statements that demonstrated the lowest scores were related to the uncertainty about the future of the job (mean = 1.82 and SD = 0.94) and the fear of losing the job soon (mean = 1.45 and SD = 0.69).

On the job resources scale, the highest scores were noted for the statements related to needing a high level of professionalism (mean = 3.82 and SD = 0.45) and learning new things (mean = 3.78 and SD = 0.46). In contrast, the lowest scores were demonstrated by the statements regarding not having sufficient time to do the work (mean = 2.25, SD = 0.84) and not having an unreasonable amount of work (mean = 2.24 and SD = 0.84), preceded by the statements regarding having the opportunity to make a lot of decisions about work (mean = 2.49 and SD = 0.77) and having a say in many things at work (mean = 2.49 and SD = 0.78).

Table 4 presents Cronbach's *α* values and Pearson's correlation coefficients of all the variables. All Cronbach's *α* values were acceptable. Moreover, burnout was moderately and positively correlated with destructive leadership and job demands but negatively correlated with job resources.

The bivariate associations of destructive leadership, job demands, and job resources with burnout were analysed in model 0 (Table 5). Background characteristics (i.e., age, gender, and work experience) that demonstrated between-group differences (Table 2) were adjusted in this model. Consequently, the association between destructive leadership and burnout was noted to be positive. Assessed as adjusted *R*^2^, destructive leadership (along with age, gender, and work experience) explained 16% of the variation in burnout. Moreover, the association between burnout and job demands was positive, explaining 11% of the variation in burnout. In contrast, job resources were negatively associated with burnout, indicating that job resources reduce burnout and explain 17% of the variation in burnout. All these associations were significant.

Multiple regression analysis was conducted for the associations of destructive leadership and job demands with burnout in model 1, and the job resources variable was added to model 2 (Table 5). The positive association of destructive leadership and job demands with burnout remained significant in model 1. Based on their adjusted *R*^2^ values, these variables explained 19% of the variation in burnout. After job resources were added, in model 2, these associations became less positive and they explained 24% of the variation in burnout overall.

## 4. Discussion

This study aimed to gain insight into the factors related to burnout in nurses. Our findings indicated that destructive leadership, as well as job demands and resources, are associated with burnout among registered nurses in Finland. To our knowledge, this is the first study to explore this multivariate association in the context of nursing. Moreover, job resources were noted to provide the strongest explanation for the variation in burnout; in particular, they were noted to be negatively correlated with burnout. Moreover, job resources were noted to diminish the negative effects of destructive leadership and job demands.

Burnout remains a major concern among nurses—as indicated by the mean exhaustion scores of 3.21 (on the 5-point Likert scale). Moreover, destructive leadership was significantly associated with burnout; therefore, destructive leadership may also be a serious threat to the well-being of nurses at work. The result was expected and is supported by previous relevant but scant research in the context of nursing [56, 57]. From the perspective of the JD-R model, destructive leadership can be considered a load factor. As a social and organisational factor, destructive leadership might lead to emotional exhaustion and a psychological withdrawal from the job [58]. Supervisors play an essential role in providing employees with job resources presented in the JD-R model (e.g., support, feedback, rewards, and various tasks) [24]. Therefore, a lack of job resources can be a result of destructive leadership. However, our results demonstrated that job resources can not only reduce burnout themselves but also mitigate the adverse effects of destructive leadership and job demands. Our results corroborate those of the JD-R model, which emphasises that job resources mitigate the negative effects of job demands [24].

Regarding the global shortage of nurses, our results indicated that the managers made the nurses feel easily replaceable, but they neither felt insecure about their job nor were they afraid about being terminated. In previous studies, nurses have reported that they felt easily replaceable, possibly because of a lack of professional respect and appreciation from their managers [59, 60]. In contrast, these feelings may occur in nurses who are temporary, from an agency or from a foreign country and have to prove their professional skills in several instances—all of which may lead to insecurity [61]. In nurses, significant associations have been noted between being temporary and vulnerability; this, in addition to some other factors, represents the feeling of being easily replaceable [62]. The amount of temporary work has increased in Nordic countries; in Finland, more than one-fifth of all nurses work under fixed-term contracts [63].

Our results also indicated that job demands are positively associated with burnout. Considering the current labour shortage, the most reported job demands include the workload and time pressure; these factors have generally been associated with increased burnout among nursing staff [64–66]. In contrast, job resources were noted to reduce burnout in the current study, which is in line with previous findings [65, 67]. In particular, in addition to job autonomy and learning opportunities [29, 67], social support and rewards are related to decreased burnout [65, 67]. The relationship between job resources and destructive leadership in nursing is unknown; however, in fields other than nursing, job autonomy has demonstrated protective effects similar to those of job resources. Job autonomy can buffer the impact of abusive supervision on factors such as job stress [68, 69]. Job crafting, which is a job resource, buffers the negative impact of abusive supervision on emotional exhaustion. The current respondents reported low job autonomy. Therefore, nurse managers should increase nurses' autonomy and decision-making opportunities, encourage them to proactively improve their work situation, and develop their job-related skills [58].

Burnout demonstrated a significant association with demographic characteristics among nurses. Notably, younger, less-experienced nurses reported the most burnout, whereas the oldest nurses demonstrated the least burnout. This result is supported by previous results [28, 70–72]. Older, more-experienced nurses might, therefore, cope better with job demands. In contrast, less-experienced nurses work longer shifts and overtime more often [73], both of which are related to higher levels of burnout [74, 75]. There might even be generational differences in work-life expectations and job resource perceptions among these age groups. For example, younger nurses report less satisfaction with the feedback and rewards they receive [76]; this feeling may be related to the feeling of not being acknowledged or respected [77]. In terms of the JD-R model, the lack of feedback and rewards are major predictors of disengagement [23]. Consequently, we suggest that these differences should be considered by supervisors from multiple generations; nurse managers should foster respectful and appreciative behaviours, especially among temporary and young nurses; they have to concentrate on building trust and giving feedback about good work and, on the other hand, guarantee professional autonomy and decision-making opportunities [78].

In the current study, men demonstrated less burnout than women. However, although previous studies have reported contradictory results, comparing their results with ours is difficult because of the differences in the burnout measures used. In some studies, compared with women, men demonstrated more depersonalisation, but this difference was not found for the other burnout dimensions [70, 79, 80]. In a study of mental health nurses, male sex was positively associated with emotional exhaustion [81]. Moreover, male transplant nurses reported significantly higher levels of personal accomplishment than their female counterparts [82]. In a meta-analysis, most studies demonstrated that women were significantly more emotionally exhausted than men [83].

The relationships between the nurses' demographic variables and destructive leadership were noted to be nonsignificant, consistent with previous findings [38]. Notably, the perception of destructive leadership is subjective and perceptions demonstrate cultural differences [41, 84–86]. Cross-cultural research on cultural differences in the relationship between burnout and destructive leadership is warranted. In addition, to develop leadership practices, the organisational context needs to be considered; social, cultural, or institutional contexts can either facilitate or constrain leadership practices [87]. Furthermore, the need for accurate data collection tools in nursing is evident; very few measurement tools to quantify different destructive leadership styles have been developed [88]. In addition, empirical research with a longitudinal, qualitative approach may aid in gaining further insight into the impact of destructive leadership.

### 4.1. Implications for Nursing Management and Practice

To improve leadership quality within the nursing profession, it is essential to recognise and acknowledge destructive leadership styles in healthcare organisations. Targeted leadership intervention programs might help focus on supportive and transformational leadership styles to reduce burnout among nurses [89]. Burnout risk factors, such as excessive job demands and inadequate resources, should be regularly assessed by management, and necessary support should be provided to nurses. The support could include well-being promotion measures focusing on resilience building, stress management, and coping strategies to mitigate the impact of destructive leadership and high job demands. Burnout prevention and stress management skills must be considered in nursing education [90]. However, the best results are obtained by investing in sustainable solutions that address structural and systemic issues and focus on creating a positive work culture. Furthermore, the effects of destructive leadership on nurses' and patients' outcomes require further examination, considering various cultural and contextual factors.

### 4.2. Strengths and Limitations

A major strength of the current study was the large, nationwide sample of registered nurses. Because the assumptions of regression were met, our findings are also generalisable. However, despite the sufficient sample size, the lack of accurate response rate calculation must be considered a limitation. Furthermore, the cross-sectional design of the study remains a limitation in this study. Therefore, the causality of relationships noted in this study should be examined further. Furthermore, the burnout-related results could not be fully compared with those from previous studies because the BAT is a relatively new measurement tool; nevertheless, most burnout questionnaires also include exhaustion as a core dimension. In this study, the Cronbach's *α* for the burnout dimension was similar to that in the original study on the BAT-4 [50]. However, further research on the relationships between the BAT and different constructs in different cultural settings [6] is warranted.

## 5. Conclusions

The current results supplement and strengthen the scant evidence on the relationship between destructive leadership and burnout among nurses. Results also confirm that among nurses, the association between job demands and burnout is positive and that job resources may alleviate the adverse effects of destructive leadership and job demands.

In healthcare organisations, strengthening job resources is essential for improving work-related well-being in terms of burnout among nurses, particularly younger nurses. To further improve the nurses' working conditions, organisations should pay attention to the evaluation of job demands and leadership styles. Through the creation of health-promoting work environments, nurse turnover may be reduced. Improved work-related well-being affects nurses' job performance, which is essential for healthcare organisations to function effectively and provide high-quality healthcare services.

The current results may be used when planning intervention studies on burnout prevention and reduction among nurses; they may also guide nurse leadership educators. However, additional studies on destructive leadership and burnout in different healthcare and cultural settings are warranted [91].

## Figures and Tables

**Table 1:** Background characteristics of the nurses (*n* = 2115).

	Mean (SD)	*n*	%
Age (years)	45.6 (10.9)		
<35		423	20.0
35–50		867	41.0
>50		782	37.0
Gender			
Male		142	6.7
Female		1956	92.5
Education level			
Vocational degree		573	27.1
Bachelor's degree		1361	64.3
Master's degree		173	8.2
Work experience (years)	17.1 (10.8)		
<5		387	18.3
5–10		327	15.5
>10		1398	66.1

**Table 2:** BAT-4 scores (overall and stratified by background characteristics, scale 1–5).

	Mean (SD)	*p*
BAT-4	2.62 (0.64)	
Exhaustion	3.21 (0.86)	
Mental distance from work	2.66 (0.95)	
Cognitive impairment	2.60 (0.83)	
Emotional impairment	2.00 (0.77)	
Age (years)		0.003
<35	2.69 (0.63)	
35–50	2.64 (0.64)	
>50	2.56 (0.64)	
Gender		0.038
Male	2.51 (0.67)	
Female	2.62 (0.63)	
Education level		0.165
Vocational degree	2.58 (0.63)	
Bachelor's degree	2.63 (0.64)	
Master's degree	2.60 (0.65)	
Work experience (years)		0.016
<5	2.60 (0.63)	
5–10	2.71 (0.62)	
>10	2.60 (0.64)	

BAT = Burnout Assessment Tool.

**Table 3:** Destructive leadership scale scores (scale 1–5).

	Mean (SD)
My bosses make me feel like I am easily replaceable	2.76 (1.35)
I am treated in an authoritarian (i.e., bossy or commanding) way	2.57 (1.23)
If I were treated unfairly, I would not dare to argue	2.26 (1.19)
If I wanted better working conditions, I would be afraid to ask	2.21 (1.10)
I am treated in an unfair or discriminatory way	2.10 (1.18)
I have to worry about being fired if I were to participate in a strike	1.67 (0.97)
I have to worry about being fired when I temporarily do not work as well	1.65 (0.92)
I have to worry about being fired if I do not immediately do what I am told	1.61 (0.91)
I am treated in an aggressive manner	1.57 (0.94)

**Table 4:** Cronbach's *α* and correlation coefficients of all the study variables.

	Burnout	Destructive leadership	Job demands	Job resources
Burnout	0.731			
Destructive leadership	0.390^*∗*^	0.897		
Job demands	0.328^*∗*^	0.425^*∗*^	0.719	
Job resources	−0.402^*∗*^	−0.450^*∗*^	−0.343^*∗*^	0.665

^*∗*^ *p* < 0.001.

**Table 5:** Linear regression models for the associations of destructive leadership, job demands, and job resources with burnout.

	Model 0		Model 1		Model 2	
	Β	SE	B	SE	B	SE
Destructive leadership	0.387^*∗*^	0.016	0.306^*∗*^	0.017	0.209^*∗*^	0.018
Job demands	0.323^*∗*^	0.026	0.193^*∗*^	0.027	0.141^*∗*^	0.027
Job resources	−0.406^*∗*^	0.036			−0.262^*∗*^	0.040
*R* ^2^			0.19		0.24	

Model 0: bivariate associations. Models 1 and 2: multivariate associations. Models are adjusted for age, gender, and work experience. B = standardised beta and SE = standard error. ^*∗*^*p* < 0.001.

## Data Availability

The survey data used to support the findings of this study have not been made available because it is part of the ongoing project.
